# Annexin A8 regulates Wnt signaling to maintain the phenotypic plasticity of retinal pigment epithelial cells

**DOI:** 10.1038/s41598-020-58296-w

**Published:** 2020-01-27

**Authors:** Katharina Lueck, Amanda-Jayne F. Carr, Lu Yu, John Greenwood, Stephen E. Moss

**Affiliations:** 1 UCL Institute of Ophthalmology, 11-43 Bath Street, EC1V 9EL London, United Kingdom; 2 PAREXEL International, The Quays, 101-105 Oxford Road UB8 1LZ, Uxbridge, United Kingdom

**Keywords:** Cell biology, Developmental biology

## Abstract

Wnt signalling mediates complex cell-cellinteractions during development and proliferation. Annexin A8 (AnxA8), a calcium-dependent phospholipid-binding protein, and canonical Wnt signalling mechanisms have both been implicated in retinal pigment epithelial (RPE) cell differentiation. The aim here was to examine the possibility of cross-talk between AnxA8 and Wnt signalling, as both are down-regulated upon fenretinide (FR)-mediated RPE transdifferentiation. AnxA8 suppression in RPE cells via siRNA or administration of FR induced neuronal-like cell transdifferentiation and reduced expression of Wnt-related genes, as measured by real-time PCR and western blotting. AnxA8 gene expression, on the other hand, remained unaltered upon manipulating Wnt signalling, suggesting Wnt-related genes to be downstream effectors of AnxA8. Co-immunoprecipitation revealed an interaction between AnxA8 and β-catenin, which was reduced in the presence of activated TGF-β1. TGF-β1 signalling also reversed the AnxA8 loss-induced cell morphology changes, and induced β-catenin translocation and GSK-3β phosphorylation in the absence of AnxA8. Ectopic over-expression of AnxA8 led to an increase in active β-catenin and GSK-3β phosphorylation. These data demonstrate an important role for AnxA8 as a regulator of Wnt signalling and a determinant of RPE phenotype, with implications for regenerative medicine approaches that utilise stem cell-derived RPE cells to treat conditions such as age-related macular degeneration.

## Introduction

*In vivo*, retinal pigment epithelial (RPE) cell phenotype is sustained by the retinal microenvironment. However, once removed from the retina and placed in culture, RPE cells dedifferentiate within a few rounds of division, losing signature characteristics such as pigment granules and expression of genes such as MerTk and RPE65. The widely used human RPE cell line, ARPE19, is typical in this respect, though several studies have shown that under appropriate culture conditions ARPE19 cells will re-adopt a more mature phenotype that includes restoration of pigment granules and expression of key RPE-associated genes^1–3^. Interest in RPE de-differentiation has also been driven by the need to understand the process in proliferative vitreoretinopathy (PVR) where epithelial mesenchymal transition (EMT) plays a key role in the pathogenesis of this condition. More recently, interest in RPE cell differentiation and maturation has intensified with advances in regenerative medicine that utilize RPE cells derived from embryonic stem (ES) cells or induced pluripotent stem (iPS) cells^4–6^. RPE cells derived from ES or iPS cells exhibit many characteristics of mature fully differentiated RPE cells, and first-in-man transplantation studies in dry and wet age-related macular degeneration (AMD) have yielded encouraging results^7–10^. Key to these clinical advances is a better understanding of the signaling pathways that regulate and maintain RPE cell phenotype.

The plasticity of RPE cells in culture is evident from studies showing that not only can they dedifferentiate, but they can also transdifferentiate. Thus, low doses of the retinoic acid (RA) derivative fenretinide (FR) inhibit RPE cell proliferation and induce a neuronal-like phenotype^11,12^. In our investigations into the mechanisms underlying the RPE response to FR, we found that FR-mediated RPE cell transdifferentiation is dependent on, and mediated by, AnxA8 downregulation^13^, demonstrating a key role for this phospholipid- and calcium-binding protein in maintaining the plasticity of the RPE cell phenotype.

A microarray analysis performed on FR-transdifferentiated RPE cells revealed down-regulation of AnxA8 and suppression of several genes involved in Wnt signaling^13^, raising the question of whether cross-talk occurs between AnxA8 and Wnt signaling. Canonical Wnt signaling maintains cell fate specification and proliferation in diverse mammalian cell types^14,15^ and it occurs upon binding of secreted Wnt proteins to Frizzled receptors and their co-receptors, lipoprotein receptor-related proteins (LRP)-5 and 6. This inactivates glycogen synthase kinase (GSK)-3β, leading to accumulation of non-phosphorylated β-catenin in the cytosol^16^. β-Catenin is then translocated to the nucleus to promote ECF/LEF-1 mediated expression of Wnt target genes. In the absence of Wnt, β-catenin is degraded by a complex consisting of GSK-3β, axin, protein phosphatase 2a, adenomatosis polyposis coli and casein kinase 1α.

Here, we report that RPE phenotype is critically dependent on canonical Wnt signaling, and that this in turn is regulated by AnxA8. We thus identify a novel signaling nexus that has implications for strategies aimed at preventing dedifferentiation and at yielding mature RPE cells from ES or iPS cells.

## Results

### FR and AnxA8 loss both induce neuronal transdifferentiation

ARPE19 cells readily dedifferentiate in culture and can be induced to transdifferentiate towards a neuronal-like phenotype upon certain stimuli^11,17^. We recently found that AnxA8 was down-regulated in ARPE-19 cells induced towards a neuronal lineage following treatment with FR, and showed that AnxA8 down-regulation is both necessary and sufficient for RPE transdifferentiation^13^. FR-induced AnxA8 loss also correlated with decreased expression of the Wnt-related genes Frizzled-1, Frizzled-4 and Wnt2b (Table 1), leading us to hypothesize that AnxA8 may regulate RPE phenotype via modulation of Wnt signaling.

**Table 1:** Microarray analysis pre- and post-FR treatment.

gene symbol	Description	fold change
ANXA8	Annexin A8	−4.63574
FZD1	Frizzled homolog 1	−1.52405
FZD4	Frizzled homolog 4	−1.61569
WNT2B	wingless-type MMTV integration site family, member 2B	−1.44753

FR treatment downregulated gene expression of AnxA8, as well as Wnt signaling-related genes Frizzled-1 (FZD1), Frizzled-4 (FZD4) and Wnt2b (WNT2B) in the human ARPE19 cell line.

### AnxA8 depletion suppresses Wnt signaling

**Figure 1:**
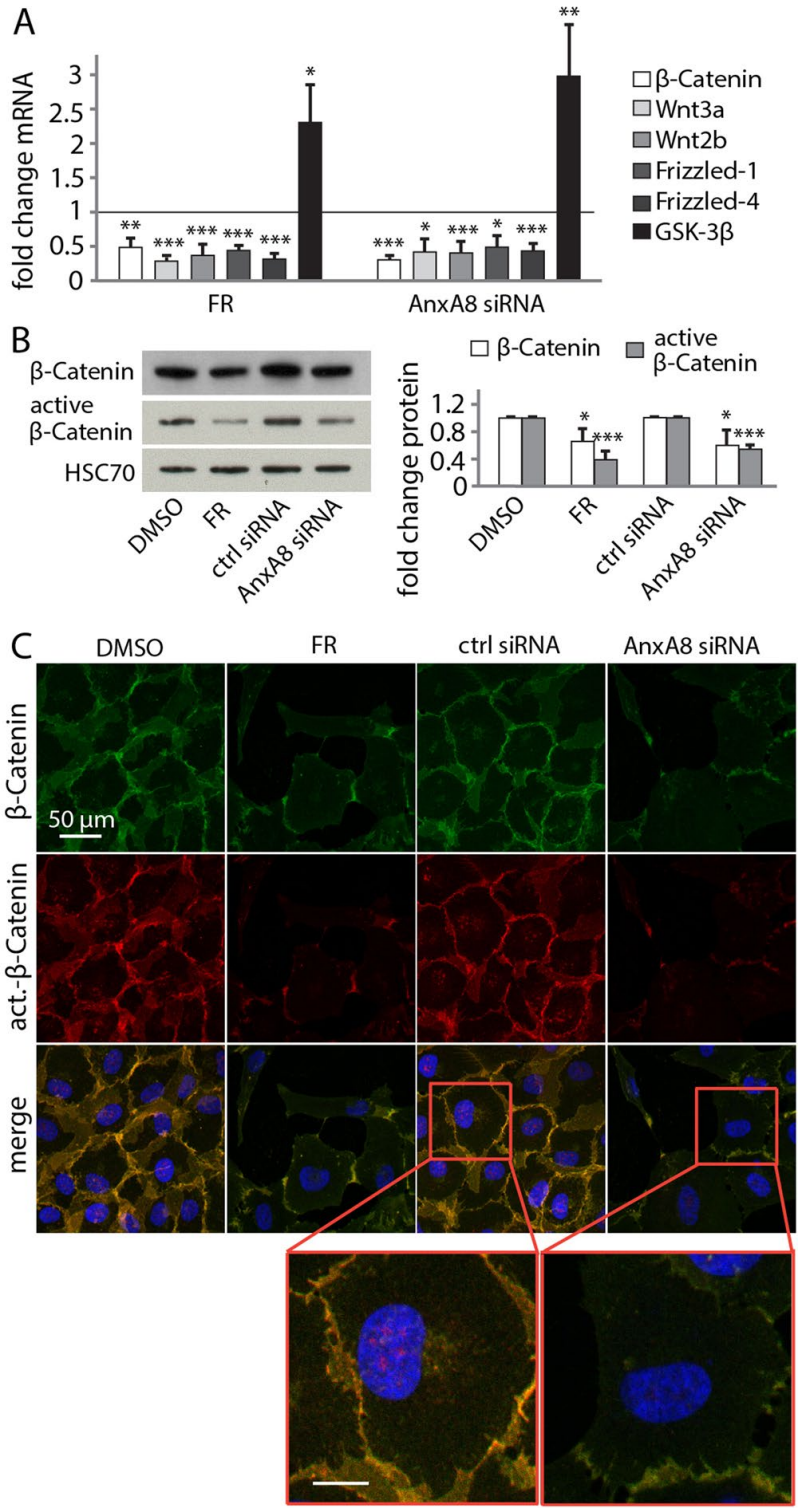
AnxA8 depletion suppresses Wnt signaling (**A**) Real-time PCR revealed decreased expression levels of Wnt signaling–related genes after exposure to FR and AnxA8 siRNA, while GSK-3β expression was increased. Shown are mean and standard deviation of five independent experiments. (**B**) Western blots showing that FR as well as AnxA8 suppression results in significantly less β-catenin and active β-catenin protein in RPE cell lysates compared to controls. Quantification was performed by densitometric analysis of 4 different data sets, and expressed as mean and standard deviation. Asterisks indicate statistical significance. *p ≤ 0.05, **p ≤ 0.01, ***p ≤ 0.001 (**C**) Immunofluorescence staining of FR- and AnxA8 siRNA-treated RPE cells displayed loss of β-catenin (green) and active β-catenin (red) at cell-cell contact sites and in the perinuclear region. Shown are representative images of three independent experiments and higher magnification images.

To test this hypothesis, we first compared the expression of Wnt-related genes in the presence of FR and following AnxA8 knockdown with siRNA. FR or AnxA8 suppression in RPE cells led to a significant decrease in mRNA levels of β-catenin, Wnt2b, Wnt3a, Frizzled-1 and Frizzled-4 (Fig. 1A). We also observed a significant reduction in both total β-catenin and active β-catenin protein after exposure to FR and AnxA8 siRNA (Fig. 1B). Consistent with this, immunofluorescence analysis revealed reduced staining at cell-cell contact sites for both β-catenin and active β-catenin in AnxA8-depleted or FR-treated RPE cells compared to controls. There was also a reduction in perinuclear staining of β-catenin and its active form (Fig. 1C). In contrast, AnxA8-depleted and FR-treated RPE cells both exhibited an increase in GSK-3β gene expression levels (Fig. 1A) that was also evident using immunofluorescence imaging (Fig. S2).

### AnxA8 gene expression is not affected by Wnt signaling

**Figure 2:**
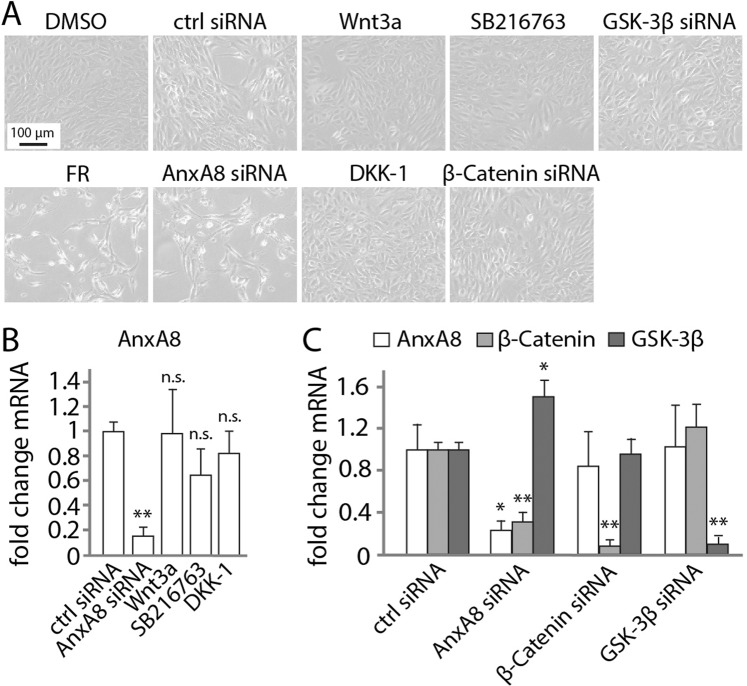
AnxA8 gene expression is not affected by Wnt signaling (**A**) Phase images reveal a normal ARPE-19 cell phenotype when Wnt signaling is activated or inhibited. Suppression of AnxA8 led to reduced proliferation and formation of extensions. Shown are representative images from 4 independent experiments (**B**) ARPE19 cells exposed to AnxA8 siRNA exhibited reduced AnxA8 gene expression, while control treatments as well as Wnt activators and inhibitor did not affect AnxA8 expression. Presented are mean and standard deviation of 4 individual data sets. Asterisks indicate statistical significance. **p ≤ 0.01 (**C**) AnxA8 suppression in ARPE19 cells induced a significant downregulation in AnxA8 and β-catenin, and an increase in GSK-3β. However, silencing β-catenin or GSK-3β suppressed expression of their respective genes, but did not change AnxA8 mRNA transcript levels. Illustrated are mean and standard deviation of 5 independent experiments determined by PCR. Asterisks indicate statistical significance. *p ≤ 0.05, **p ≤ 0.01.

Having shown that AnxA8 can influence the expression of genes involved in Wnt signaling we next asked whether the reverse occurs, do changes in canonical Wnt signaling influence AnxA8 expression? Recombinant Wnt3a and the GSK-3β inhibitor SB216763 were used to activate Wnt signaling in culture, while DKK-1 was used to inhibit Wnt signaling. Phase images revealed that cell growth and morphology were indistinguishable in control-treated ARPE19 and primary porcine RPE (pRPE) cells, and in cells treated with Wnt signaling activators or inhibitor (Figs. 2A and S3A). Furthermore, real-time PCR analysis revealed no significant difference in AnxA8 expression between untreated RPE cells and those treated with Wnt activators or inhibitor (Figs. 2B and S3B). Using a different approach, siRNA-mediated suppression of β-catenin or GSK-3β similarly had no effect on AnxA8 expression in RPE cells whereas, consistent with the data in Fig. 1, AnxA8 siRNA led to down-regulation of both AnxA8 and β-catenin gene expression, and up-regulation of GSK-3β (Figs. 2C and S3C).

### AnxA8 physically associates with β-catenin

**Figure 3:**
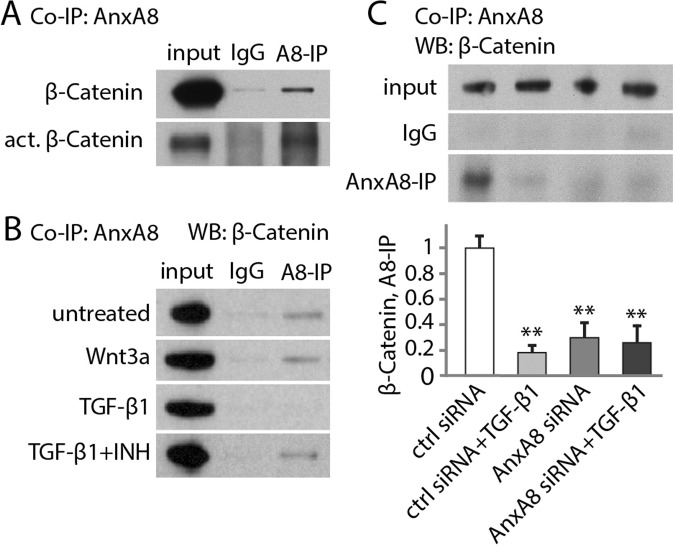
AnxA8 physically associates with β-catenin. (**A**) ARPE19 cells were subjected to immunoprecipitation with anti-AnxA8 antibody followed by immunoblotting with β-catenin and active β-catenin. Shown are representative western blots from 4 independent experiments. (**B**) Pre-exposure of cells to Wnt3a for 1 hour left the interaction unaltered, however, stimulation with TGF-β1 disrupted the interaction, though this was restored when counteracting TGF-β1 signaling with the Alk5 inhibitor SB431542 (INH). Experiments were repeated 3–5 times. (**C**) The AnxA8/β-catenin interaction decreased significantly in the presence of TGF-β1, and in a control in which AnxA8 was depleted using siRNA. Shown are representative immunoblots and the statistical values are plotted in a graph as mean and standard deviation. Significant changes are marked with asterisks. **p ≤ 0.01.

To investigate possible physical association between AnxA8 and components of the Wnt signalosome, we performed co-immunoprecipitation experiments and observed interactions between AnxA8 and both β-catenin and active β-catenin in ARPE19 cells (Fig. 3A). The interaction between AnxA8 and β-catenin was unaffected by exposure to Wnt signaling activators and inhibitors, Wnt3a or DKK-1 respectively. However, TGF-β1, which drives nuclear translocation of β-catenin, disrupted the interaction between β-catenin and AnxA8. In contrast, blocking TGF-β1 signaling with the Alk5 inhibitor SB431542 restored the interaction (Fig. 3B). AnxA8 depletion also led, as expected, to the loss of the interaction between AnxA8 and β-catenin (Fig. 3C).

### AnxA8 depletion restricts canonical Wnt signalling

**Figure 4:**
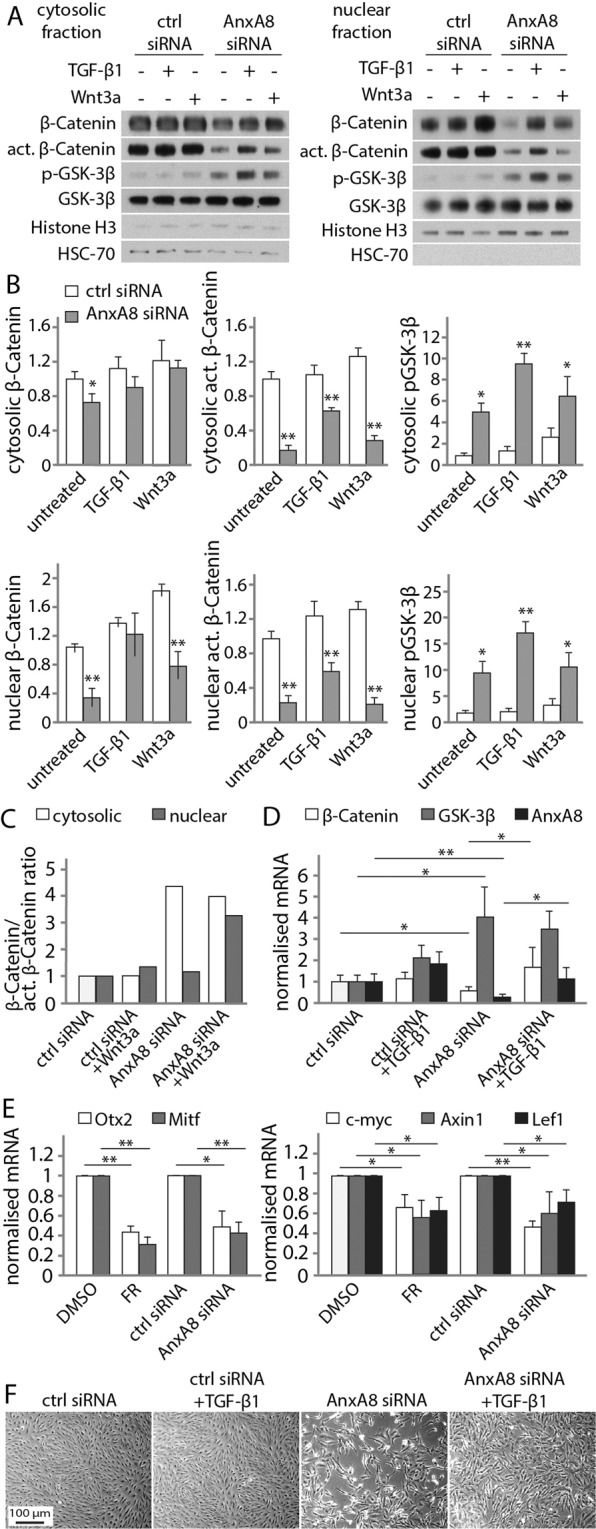
AnxA8 depletion restricts canonical Wnt signalling. (**A**) Analysis of cytosolic and nuclear fractions of RPE cells revealed a decrease in β-catenin and active β-catenin protein expression as well as nuclear translocation upon AnxA8 suppression. TGF-β1 was capable of restoring β-catenin and its active form in the absence of AnxA8. GSK-3β protein levels remained unchanged, however, GSK-3β was phosphorylated in the absence of AnxA8 and more so in combination with TGF-β1. Shown are representative western blots of 4 individual experiments. (**B**) Densitometric analysis of the blots shown in (**A**). Statistics are expressed as mean and standard deviation, and asterisks indicate statistical significance. *p ≤ 0.05, **p ≤ 0.01. (**C**) β-catenin/active β-catenin ratio analysed from the statistical data from (**B**) revealed a shift towards β-catenin in nuclear fractions of AnxA8-depleted cell treated in response to Wnt3a. (**D**) RPE cells treated with AnxA8 siRNA revealed reduced β-catenin, AnxA8, and elevated GSK-3β mRNA transcripts analysed by PCR. TGF-β1 is partly capable of restoring β-catenin and AnxA8 expression levels in AnxA8-depleted cells. (**E**) Transcripts of Wnt-dependent RPE transcription factors Mitf and Otx2, as well as canonical Wnt target genes Axin1, c-myc and Lef1 were decreased upon treatment with FR or AnxA8 siRNA. Illustrated are mean and standard deviation of at least 4 separate data sets. Asterisks indicate statistical significance. *p ≤ 0.05, **p ≤ 0.01. (**F**) Phase images show formation of extensions and arrested proliferation in AnxA8-depleted cells. Exposure to TGF-β1 in the absence of AnxA8 resulted in relatively normal ARPE-19 cell growth. Depicted are representative images from 4 individual experiments.

We next investigated how interactions between AnxA8, β-catenin and GSK-3β influence Wnt signaling. We therefore examined cytosolic and nuclear pools of β-catenin and GSK-3β in the presence and absence of siRNA for AnxA8, and with or without cell stimulation with Wnt3a and TGF-β1 in human ARPE19 (Fig. 4) and in primary porcine RPE cells (Fig. S4). In these experiments, histone H3 and HSC-70 were used as nuclear and cytosolic markers respectively (Fig. 4A). Suppression of AnxA8 led to a small decrease in cytosolic β-catenin and a more substantial decrease in its active form, with expression of the latter being partially reversed by TGF-β1 but not Wnt3a. Nuclear β-catenin and its active form, both of which were markedly reduced in the absence of AnxA8, were also partially restored by TGF-β1. Total GSK-3β protein was present in both cytosolic and nuclear fractions, with elevated cytosolic levels in the absence of AnxA8, consistent with the data in Fig. 1A. Phosphorylation of GSK-3β was barely detectable in control conditions, with only Wnt3a inducing slight GSK-3β phosphorylation. However, we observed significantly increased phosphorylation of cytosolic (5 fold) and nuclear (10 fold) GSK-3β upon AnxA8 suppression, which was further increased by TGF-β1 (Figs. 4A,B and S4A,B). Analysis of the β-catenin/active β-catenin ratio revealed a shift towards β-catenin in the nuclear fractions of AnxA8-depleted cells in response to Wnt3a (Fig. 4C). qPCR analysis of AnxA8-suppressed RPE cells confirmed down-regulation of AnxA8 and β-catenin, with concomitant up-regulation of GSK-3β. Upon exposure to TGF-β1, there was an elevation in AnxA8 gene expression, while GSK-3β was unaltered, and β-catenin mRNA levels remained unchanged. Activating TGF-β1 in AnxA8-suppressed ARPE19 cells did not impact on AnxA8 and β-catenin expression levels, and GSK-3β mRNA transcripts in AnxA8-depleted cells were unaffected by TGF-β1 (Fig. 4D). Active TGF-β1 signaling and AnxA8-suppression both restored the expression of β-catenin and reversed the AnxA8 loss-induced transdifferentiation of RPE cells towards a neuronal-like phenotype (Fig. 4F). Thus, while suppression of AnxA8 resulted in growth arrest and formation of cell extensions in both ARPE19 and primary RPE cells, TGF-β1 restored normal growth morphology in AnxA8-reduced cells (Figs. 4F and S4C). Concomitant with the decrease in β-catenin, the Wnt-dependent RPE transcription factors microphthalmia-associated transcription factor (Mitf) and orthodenticle homolog 2 (Otx2), as well as the canonical Wnt target genes c-myc, Axin1 and LEF1 were also decreased after depletion of AnxA8 (Fig. 4E).

### Overexpression of AnxA8 amplifies canonical Wnt signaling

**Figure 5:**
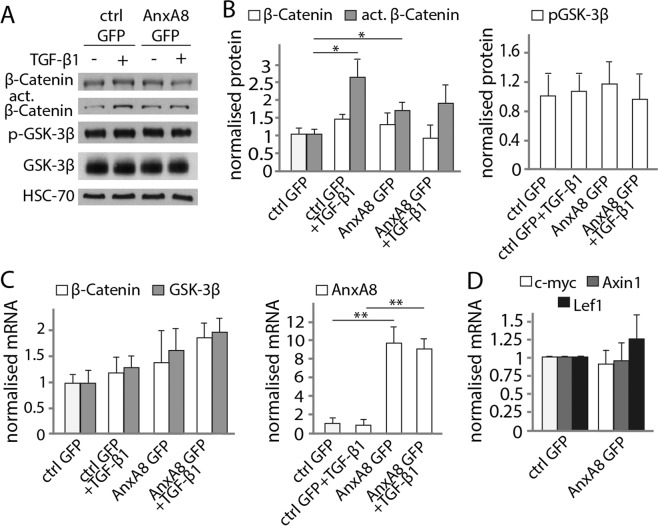
Overexpression of AnxA8 amplifies canonical Wnt signaling. (**A**) Western blotting reveals an increase in active β-catenin and GSK-3β protein in AnxA8-GFP transfected cells compared to controls, while β-catenin is unchanged (**B**) Quantification of blots such as those in A are presented as mean and standard deviation. Significant changes are marked with asterisks. *p ≤ 0.05. (**C**) AnxA8 overexpression in ARPE19 cells yielded increased levels of AnxA8 and slightly higher levels of GSK-3β mRNA. Addition of TGF-β1 led to increased β-catenin expression levels in AnxA8-overexpressing cells. Values are mean and standard deviation from 4 independent experiments determined by PCR. Asterisks indicate statistical significance. **p ≤ 0.01. (**D**) Transcripts of canonical Wnt target genes Axin1, c-myc and Lef1 were not significantly altered upon overexpression of AnxA8. Illustrated are mean and standard deviation of 4 separate data sets.

We then over-expressed AnxA8 in RPE cells using an AnxA8-GFP construct to examine Wnt signaling in the presence of exogenous AnxA8. Transient transfection with AnxA8-GFP led to increased levels of active β-catenin protein. However, total β-catenin levels were unchanged and there was no change in the GSK-3β phosphorylation status. TGF-β1 signaling induced an increase in active β-catenin only (Fig. 5A,B). As expected, AnxA8 mRNA transcripts were significantly increased in AnxA8-GFP transfected RPE cells, independent of TGF-β1 signaling activity (Fig. 5C). The expression of canonical Wnt target genes c-myc, Axin1 and Lef1 was not changed in RPE cells over-expressing AnxA8 (Fig. 5D).

## Discussion

In this study and previous work, we have shown that in RPE cells, FR-induced neuronal transdifferentiation is mediated at least in part by suppression of AnxA8^13^, consistent with roles for AnxA8 in the phenotypic regulation of other cell types^18,19^. Here we found that exposure of RPE cells to FR also led to down-regulation of several key genes involved in canonical Wnt signaling, including β-catenin, Wnt2b, Wnt3a, Frizzled-1 and Frizzled-4, whereas in contrast GSK-3β expression was raised. RA derivatives are well known to suppress canonical Wnt signaling in different cancer cell lines by destabilization of β-catenin^20,21^. There is also a positive correlation between cell differentiation and increased GSK-3β-mediated degradation of phosphorylated β-catenin in the presence of all-trans RA^22,23^. Interestingly, we observed an almost identical shift in Wnt-related genes in AnxA8-depleted RPE cells, suggesting that AnxA8 is required for the maintenance of Wnt signaling in RPE cells.

Here we observed a decrease in Wnt gene expression in cultured RPE cells within two days of applying AnxA8 siRNA (not shown), and after four days cells adopted neuronal-like cell morphology. Canonical Wnt signaling appears to have phased effects on neuronal differentiation. Thus, Wnt signaling is suppressed during the initial phase of neuronal differentiation^24^, but is elevated during the latter stages. Demonstrating that AnxA8 is required to maintain Wnt signaling prompted us to ask whether there is a reciprocal interdependency in which β-catenin or GSK-3β may similarly be required for AnxA8 expression. However, whilst depletion of AnxA8 led to down-regulation of Wnt signaling, we found that neither stimulation nor inhibition of Wnt signaling had any impact on RPE morphology or AnxA8 expression in ARPE19 and primary porcine RPE cells, placing AnxA8 activity upstream of Wnt signaling.

To understand how AnxA8 exerts its influence over Wnt signaling, we asked whether it interacts with components of the Wnt signaling machinery, and observed that AnxA8 physically associates with β-catenin. Several studies have shown that cross-talk between TGF-β signaling and Wnt signaling pathways plays an important role in the regulation of certain developmental events, and here we observed that the AnxA8/β-catenin association was reduced when RPE cells were exposed to TGF-β1. The cooperative regulation by Wnt signaling and TGF-β1 is mediated by an association between Smads and TCF/LEF in the nucleus, and results in the synergistic activation of specific target genes^25,26^. TGF-β1 has been shown to stimulate nuclear translocation of β-catenin^27^, and to upregulate several Wnts and thereby stabilise β-catenin^28^. TGF-β1 further seems to be capable of controlling osteoblast differentiation in mouse C2C12 cells^29^.

Here we have shown that AnxA8 functions as a regulator of canonical Wnt signaling in RPE cells. Suppression of AnxA8 led to a significant decrease in the key Wnt signaling protein β-catenin and its active form in both cytosol and nucleus. Concomitantly, increasing phosphorylation of GSK-3β at serine-9 leads to a reduction in GSK-3β-mediated phosphorylation of β-catenin, and therefore to an activation of Wnt signaling^30^. The cytoplasmic accumulation of β-catenin and its nuclear translocation have consequences for gene expression, which in some cases have been implicated in pathological conditions such as AMD^31^. In AnxA8-depleted RPE cells, we observed a decrease in several β-catenin target genes, including Otx2 and Mitf, two major transcription factors with roles in RPE development. In the context of PVR, Han and colleagues found that laser photocoagulation activates Wnt signaling, leading to increased RPE proliferation and epithelial to mesenchymal transition through elevated Otx2 and Mitf^32^.

TGF-β1 signaling has been shown to increase β-catenin expression and nuclear translocation in various cell types^33^, but here we observed only marginal increases in β-catenin upon exposure of RPE cells to TGF-β1. However, cells depleted of AnxA8 showed a significant increase in β-catenin and active β-catenin when stimulated with TGF-β1. We also observed an increase in the phosphorylation of GSK-3β in TGF-β1-treated AnxA8-suppressed RPE cells. This is consistent with reports that TGF-β1 increases the expression of the ser9-phosphorylated inactive form of GSK-3β in human lung fibroblasts^27^. We also found that TGF-β1 blocked AnxA8 siRNA-induced neuronal transdifferentiation, which is in line with data describing that inhibition of GSK-3β suppresses RA-mediated differentiation of ES cells^34^. TGF-β1 was reported to stimulate a physical association between GSK-3β and Smad3, thereby facilitating β-catenin translocation^35,36^. However, it is well known that TGF-β1 signaling is highly context-dependent. It can induce Wnt signaling in pulmonary fibroblasts^37^ and inhibit photoreceptor differentiation through down-regulation of the canonical Wnt pathway in human Müller glia^38^.

In summary, we have identified a novel role for AnxA8 in the regulation of RPE phenotype that is mediated through canonical Wnt signaling. This is consistent with the observation in the regenerating chick retina that β-catenin prevents RPE cells from entering the cell cycle, whereas loss of β-catenin leads to dedifferentiation and proliferation^39^. Wnt signaling also participates in ES cell differentiation towards RPE cells^40^ where it seems to control self-renewal and spontaneous differentiation of naïve human ES cells. Decreased Wnt signaling activity was found to inhibit differentiation of ES cells^41,42^, suggesting that AnxA8-mediated changes in Wnt signaling might potentially have an impact on stem cell fates. A better understanding of the interplay between AnxA8 and Wnt signaling could be important in the development of stem cell-based regenerative therapies for RPE replacement in diseases such as AMD.

## Materials and Methods

### Cell culture

The human ARPE-19 cell line (ATCC number CRL-2302)^43^ as well as primary pRPE were cultured in Dulbecco’s modified Eagle’s medium (DMEM, Gibco). Media was substituted with 100 U/ml penicillin/streptomycin (P/S, Gibco), 2 mM L-glutamine (Gibco) and 10% foetal bovine serum (FBS, Gibco). Incubators were kept at 37 °C and 5% CO_2_.

### FR treatment

RPE cells were plated at 2,200 cells/cm^2^ and treated with 1 µM FR (Tocris, Bioscience) or vehicle (0.1% dimethyl sulfoxide; DMSO) in DMEM and 1% FBS for 7 days each day. Phase images were taken (Leica DC200) and cells were processed for gene and protein analysis.

### Suppression of AnxA8 gene expression

RPE cells were plated and transfected on the following day at 80% confluent using Interferin small interfering ribonucleic acid (siRNA) transfection reagent (Polyplus transfection). 150 pM of the following AnxA8 siRNA sequences were used: humanAnxA8 siRNA-1, 5′-CAG CCU UUC GGU CUU CUA UTT-3′; humanAnxA8 siRNA-2, GCG UGA UGG GAC CCU GAU ATT-3′; humanAnxA8 siRNA-3, 5′-GCC CUU AUG UAC CCG CCA UTT-3′^44^. β-catenin (sc-29209, Santa Cruz) and GSK-3β siRNA (sc-35527, Santa Cruz) were used at 10 nM. Allstars negative control siRNA (Qiagen) was used at respective concentrations accompanying each experiment. RPE cells were transfected in P/S-free media for 48 h, before the transfection mix was replaced with full media for another 48 h. Phase images were taken, and cells were processed for real-time PCR (see Table 2 for primer sequences), Western blotting and immunofluorescence analysis.

**Table 2:** Human and porcine primer pairs used in real-time PCR.

Target		Sequence from 5′ to 3′
human AnxA8	F	TGG GAC CCT GAT AAG AAA CAT
	R	TCC TGG AGA CTC TGG CTT CAT
human β-Catenin	F	GCC TGC CAT CTG TGC TCT TC
	R	ACT AGT CGT GGA ATG GCA CC
human Frizzled-1	F	ATT TGG TCA GTG CTG TGC TG
	R	TCA TGA AGA GGA TGG TGC AG
human Frizzled-4	F	GGG CAC GAG CTG CAG ACG GAC G
	R	GCA CCT CTT CAT CAC CTG GCC C
human Wnt2b	F	CCG CTG TGG TCG CAC GGC TGT G
	R	AGT GCC TAG GGA ACC TGC AGC C
human Wnt3a	F	CTG TTG GGC CAC AGT ATT CC
	R	ATG AGC GTG TCA CTG CAA AG
human Otx2	F	GAC CAC TTC GGG TAT GGA CT
	R	TGG ACA AGG GGA TCT GAC AGT
human Mitf	F	GTG CCA ACT TCT TTC ATC A
	R	ACC TAA ACC GTC CAT TCA
human GAPDH	F	ACC CAC TCC TCC ACC TTT G
	R	CTC TTG TGC TCT TGC TGG G
human GSK-3β	F	GCG GAG AGC TGC AAG CCG G
	R	CTT GTG GCC TGT CTG GAC CC
human Axin2	F	ACT GCC CAC ACG ATA AGG AG
	R	CTG GCT ATG TCT TTG GAC CA
human LEF-1	F	CTT TAT CCA GGC TGG TCT GC
	R	TCG TTT TCC ACC ATG TTT CA
human c-myc	F	CTT CTC TCC GTC CTC GGA TTC T
	R	GAA GGT GAT CCA GAC TCT GAC CTT
porcine AnxA8	F	CCC AGA CCC CGA CGC GGA GAC C
	R	GTT CTT GGT CCG AGA AGC CAG G
porcine GAPDH	F	AAG TGG ACA TTG TCG CCA TC
	R	TCA CAA ACA TGG GGG CAT C
porcine β-Catenin	F	GCA ATG ACT CGC GCT CAG AGG
	R	CTG AGG AGA ACG CAT GAT GGC
Porcine GSK-3β	F	GGA TGG CAG CAA GGT GAC CAC
	R	CCG GAA CAT AGT CCA GCA CCA G

Shown are forward (F) and reverse (R) sequences of the primers.

### Cycloheximide (CHX) assay

CHX (30 µg/ml) was added to ARPE-19 cells treated with AnxA8 siRNA and FR at 6, 4, 2 and 0 hours before end of treatment. Cells were processed for western blotting as described in the Supplementary Information.

### Wnt activation/inhibition in culture

To analyse whether Wnt signalling has an effect on AnxA8 expression, Wnt activators or inhibitor, respectively, were added to ARPE19 and to pRPE cells. 10 nM Wnt3a (Abcam) or 2.5 µM SB216763 (Tocris Bioscience) were added every other day for 7 days to activate Wnt signalling, while 50 ng/ml DKK-1 (Life Technologies) was added 1 hour prior to FR treatment to inhibit Wnt signalling. Phase images were taken, and cells were processed for real-time PCR and Western blotting.

### Co-immunoprecipitation

Subconfluent ARPE19 cells were harvested in 1 ml of ice-cold lysis buffer (20% glycerol, 20 mM Tris-HCl pH 8.8, 150 mM NaCl, 1% NP-40 and protease inhibitors (Complete) on ice for 30 min. The lysate was spun down at 10,000 g at 4 °C for 15 min, and the supernatant was incubated with 50 µl Dynabeads Protein A (Life Technologies) for 30 minutes at 4 °C. Beads were pelleted at 2500 g for 3 minutes at 4 °C and an aliquot of the pre-cleared lysate was stored at −20 °C. 5 µg of rabbit anti-AnxA8 (Avira) or equivalent amounts of rabbit IgG (Sigma) was added to the pre-cleared lysate and incubated for 1 hour at 4 °C. Beads were added and incubated for an additional hour. Beads were centrifuged at 2500 g for 1 min and washed with lysis buffer 5 times. Beads were diluted in 50 µl of sample buffer containing 2% sodium dodecyl sulfate (SDS), 25% glycerol, 12.5% 0.5 M Tris, 0.02% dithiothreitol and 0.2% bromophenol blue, and analysed for β-catenin using Western blotting analysis.

### Nuclear and cytoplasmic fraction

Nuclear and cytoplasmic protein levels were analysed according to Jian *et al*. (35). Briefly, cells were lysed on ice with a cytoplasmic lysis buffer (10 mM Hepes, 1.5 mM MgCl_2_, 10 mM KCl, 0.5 mM DTT, 300 mM sucrose, 0.1% NP-40, 10 mM NaF, 20 mM ß-glycerophosphate, 10 mM Na_3_VO_4_, protease inhibitors). After 10 min on ice, cells were spun down for 15 sec. The supernatant was diluted in sample buffer, whereas the pellet was washed twice in cytoplasmic lysis buffer. The pellet was then lysed in nuclear lysis buffer (50 mM Hepes, 250 mM KCl, 0.1 mM EDTA, 0.1 mM EGTA, 0.1% NP-40, 0.1% glycerol, 10 mM NaF, 10 mM Na_3_VO_4_, 1 mM DTT, protease inhibitors) for 30 min on ice. After centrifuging the nuclear proteins for 20 min at 14,000 rpm at 4 °C, the supernatant was diluted in sample buffer. Samples were boiled for 5 min and subjected to SDS-PAGE.

### Transfection with AnxA8-GFP

Subconfluent ARPE19 cells were transfected using Lipofectamine LTX Plus transfection reagent (Life Technologies). According to the manufacturer’s instructions, 2.5 µg AnxA8-GFP or GFP plasmid DNA was used to transfect a dish of 8.55 cm² for 5 h at 37 °C. Cells were washed and cultured in 10% FBS-containing DMEM for another 48 h. TGF was added in serum-free DMEM for 24 h, and cells were harvested for Western blot anaylsis.

### Statistical analysis

Data were presented as mean and standard deviations. Student T-test was performed for normally distributed data to analyse differences between two groups (Excel). One-way ANOVA and Tukey posthoc were applied for normally distributed data to evaluate differences between more than two groups. Statistically significant differences were marked with an asterisk (*).

## Supplementary information

Supplementary information.
