# Supplementary material for: Annexin A8 regulates Wnt signaling to maintain the phenotypic plasticity of retinal pigment epithelial cells
Source: Sci Rep. 2020 Jan 27;10:1256. doi: 10.1038/s41598-020-58296-w (PMC6985107; doi:10.1038/s41598-020-58296-w)
Supplement: Supplementary file 1 — Supplementary information. [file 41598_2020_58296_MOESM1_ESM.docx]

**Annexin A8 regulates Wnt signaling to maintain the phenotypic plasticity of retinal pigment epithelial cells**

**Katharina Lueck^1^, Amanda-Jayne F. Carr^1^, Lu Yu^2^, John Greenwood^1^, Stephen E. Moss^1^***

**Supplementary information**

*Supplementary Materials*

**Isolation of primary pRPE**

Pig eyes were obtained from ‘Blixes Farm’ (Chelmsford) and kept on ice during transport and dissection. The eyes were cleaned from muscle tissue, disinfected with videne (Ecolab) and incubated in 0.2 mg/ml P/S for 30 min at 4°C. Under sterile conditions, the eyes were circularly cut underneath the *Ora serrata*. The neuroretina was detached from the underlying RPE, and cut at the optic nerve. RPE cells were incubated with 10x trypsin-EDTA (Gibco) for 20 min at 37°C, spun down at 400 g for 5 min, and seeded in 6-well plates (Nunc).

**Real-time PCR**

RNA was isolated using the RNeasy Mini Kit (Qiagen), and RNA concentration was measured with Nanodrop 1000 Spectrophotometer (Thermo Scientific). Up to 1 µg RNA was transcribed into cDNA using the Quantitect RT Kit (Qiagen). Real-time amplification was performed on a HT7900 Fast Real-Time PCR System (Applied Biosystems). Power Sybr Green master mix (Life Technologies), 1 µl cDNA and 0.2 µM gene-specific primers (Life Technologies, Table 2) were combined in a PCR mix. Raw data from triplicates were transferred into the Dart-PCR spreadsheet. The gene expression was normalised to Glyceraldehyde-3-phosphate dehydrogenase (GAPDH), and dissociation curves were generated to validate primer specificity.

**Immunofluorescence staining**

Cells grown on coverslips were fixed in 4% paraformaldehyde (PFA) for 20 min at 4°C, and incubated in 0.1% Triton X100 (Sigma) for another 20 min to permeabilise the membrane. Unspecific binding sites were blocked with 1% BSA in PBS for 30 min. Primary antibodies for mouse β-Catenin (1:200, BD Biosciences), rabbit active β-Catenin (1:200, D13A1 Cell Signalling), and mouse GSK-3β (1:200, BD Biosciences) were incubated with the cells for 2 hours at RT or overnight at 4°C. Secondary Alexa flour 488 donkey anti-rabbit and 555 donkey anti-mouse antibodies (1:200, Invitrogen) were applied for 1 hour at RT. Nuclei were stained with DAPI (1:1000, Sigma) for 2 min at RT, and cells were mounted in Mowiol (Calbiochem). The slides were analysed by confocal microscopy (Leica TCS SP2), and images were processed in ImageJ (NIH freeware).

**Western Blotting**

Cells were lysed in sample buffer at 95°C for 5 minutes. Proteins were separated by SDS-polyacrylamide gel electrophoresis (SDS-PAGE) at 150 V and transferred to a nitrocellulose membrane at 35 V for 3 hours. Unspecific binding sites were blocked with 5% milk in TBS-Tween 0.05% for 30 minutes. The membrane was probed with mouse anti-heat shock chaperone 70 (HSC70, 1:5000, Santa Cruz), mouse anti-β-Catenin (1:5000), rabbit anti-active (non-phospho) β-Catenin (1:1000), rabbit anti-pGSK-3β (1:1000, Novus Biologicals), mouse GSK-3β (1:2000, BD Biosciences), and Histone H3 (D1H2, Cell Signalling) at 4°C overnight. A secondary donkey antibody directed against goat, mouse, or rabbit (1:2000, Dako) was applied for 1 hour. Bands were visualised by chemiluminescence (ECL, Amersham) and exposed to x-ray film. Densitometric analysis was performed with ImageJ (NIH freeware). Data were normalised to HSC-70, and values of untreated cells were set to 1.

*Supplementary figures*


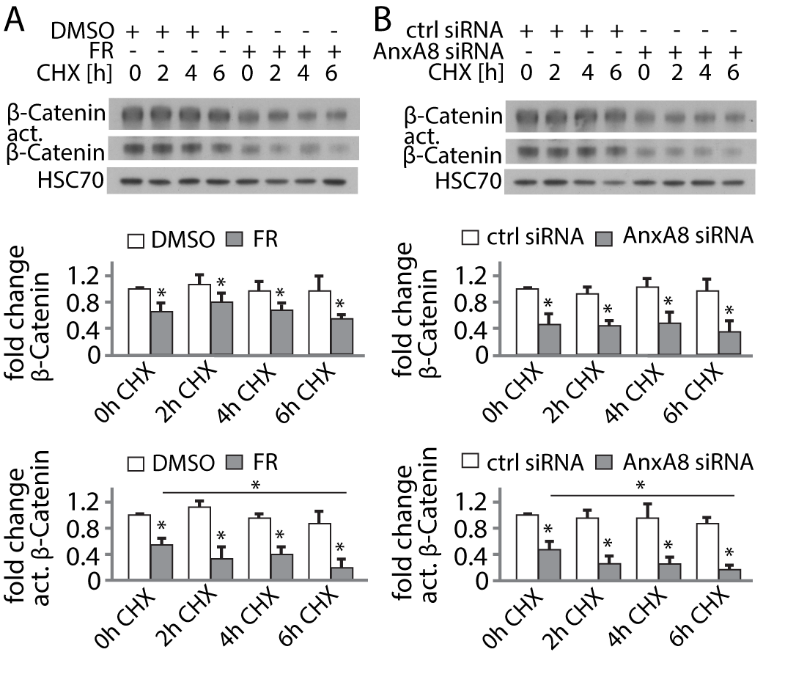


**Fig. S1: AnxA8 affects the stability of active β-Catenin**. β-catenin protein levels are lower in (A) FR- and (B) AnxA8 siRNA-treated ARPE19 cells compared to controls, but protein levels remain stable over 6 h in CHX. However, active β-catenin is significantly decreased after 6 h of CHX in both (A) FR-treated and (B) AnxA8-depleted cells. Shown are representative images as well with mean and standard deviation from 4 individual western blot experiments. Asterisks indicate statistical significance. *p ≤ 0.05.


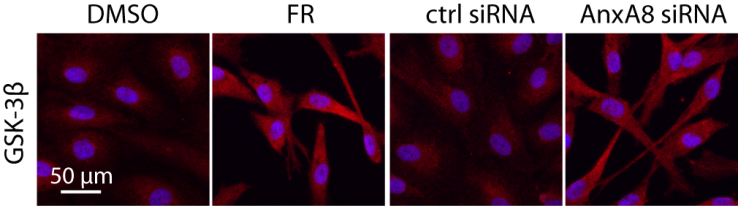


**Fig. S2: GSK-3β is increased in AnxA8-depleted RPE cells**. Immunofluorescence analysis shows increased staining for GSK-3β (red) in ARPE-19 cells after exposure to FR or AnxA8 siRNA. Nuclei were counterstained with DAPI (blue). Shown are representative images from 3 individual experiments


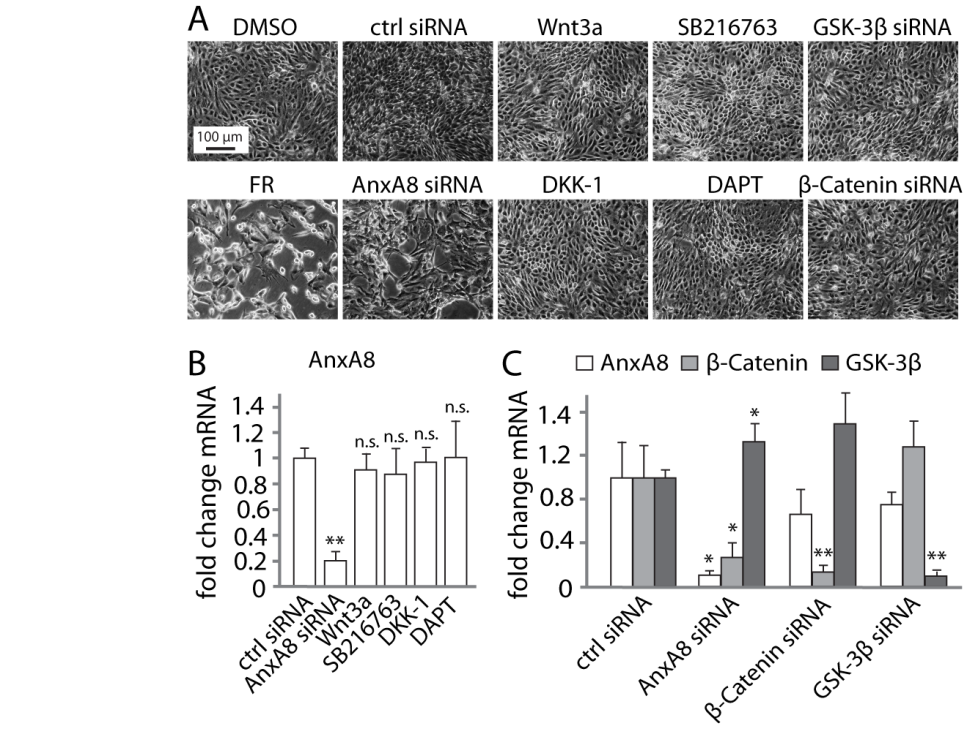


**Fig. S3: AnxA8 gene expression is unaffected by Wnt signaling.** (A) Phase images show normal cell growth of primary RPE cells after activating or inhibiting Wnt signaling. Suppression of AnxA8 led to decreased proliferation and formation of extensions. Depicted are representative images from 4 individual experiments (B) PCR analysis of primary RPE cells exposed to AnxA8 siRNA shows diminished AnxA8 gene expression, while control treatments as well as Wnt activators and inhibitor had no effect. Presented are mean and standard deviation of 3 individual data sets. Asterisks indicate statistical significance. **p≤0.01 (C) AnxA8 suppression in primary RPE cells induced significant downregulation of AnxA8 and β-catenin mRNA transcripts, and an increase in GSK-3β. However, silencing β-catenin or GSK-3β only decreased their respective targets, but did not change AnxA8 mRNA transcript levels. Illustrated are mean and standard deviation of 5 independent experiments determined by PCR. Asterisks indicate statistical significance. *p≤0.05, **p≤0.01.


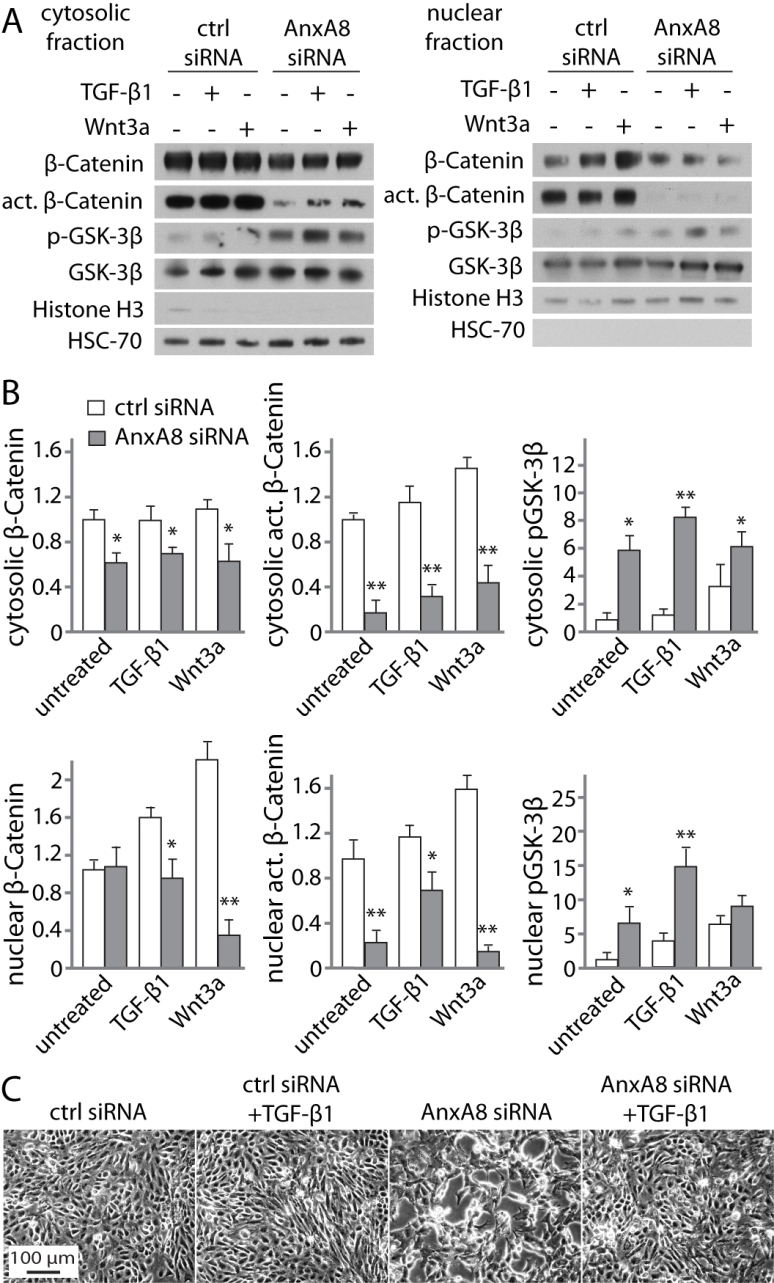


**Fig. S4:** **AnxA8 depletion reduces canonical Wnt signalling in primary RPE cells.** (A) Cytosolic and nuclear fractionation of pRPE cells yealded decreased β-catenin and active β-catenin protein upon western blotting, as well as nuclear translocation upon AnxA8 suppression. TGF-β1 was able to restore β-catenin protein levels and its active form in the absence of AnxA8. GSK-3β was phosphorylated in the absence of AnxA8 and even more in combination with TGF-β1. Shown are representative western blots of 4 individual experiments. (B) Quantitation of western blots by densitometric scanning is presented as mean and standard deviation. Asterisks indicate statistical significance. *p≤0.05, **p≤0.01. (C) Phase images reveal formation of extensions and arrested proliferation in AnxA8-depleted cells. Exposure to TGF-β1 in the absence of AnxA8 restores normal RPE cell growth. Shown are representative images from 4 individual experiments.
